# Is YouTube Reliable to Teach Laparoscopic Gastric Bypass?

**DOI:** 10.7759/cureus.62510

**Published:** 2024-06-17

**Authors:** Ahmet Tarik Harmantepe, Alp Ömer Cantürk

**Affiliations:** 1 Gastroenterology Surgery, Sakarya University Training and Research Hospital, Sakarya, TUR; 2 General Surgery, Sakarya University Training and Research Hospital, Sakarya, TUR

**Keywords:** education, gastric bypass, reliable, lrygb, youtube®

## Abstract

Aim: The increasing prevalence of obesity has led to the popularity of bariatric surgery. Laparoscopic Roux-en-Y gastric bypass (LRYGB) is one of the most complex methods in bariatric surgery. The main steps of LRYGB were determined in the Delphi Consensus. This study investigated the instructiveness and reliability of YouTube videos about LRYGB based on the Delphi Consensus.

Methods: In February 2024, three different searches were done in the search bar of the YouTube platform with the terms "laparoscopic gastric bypass" "laparoscopic Roux-en-Y gastric bypass" and "laparoscopic RYGB". The first 50 videos in each search were evaluated. Animations, lectures, advertisements, non-English videos, and non-surgical videos (pre-surgery, post-surgery vlog, etc.) were excluded from the study. Delphi consensus steps were used to determine the reliability of the videos. The quality of the videos was measured using the Global Quality Scale (GQS) and modified DISCERN test.

Results: Forty-five videos were included in the evaluation. While 14 (31.1%) of these videos were classified as reliable, 31 (68.8%) were not found reliable. In reliable videos, video description, high definition (HD) resolution, GQS, and modified DISCERN were significantly higher (p-value 0.023, 0.004, 0.017, and 0.025 respectively).

Conclusion: The rate of unreliable videos was higher on the YouTube platform. We conclude that YouTube alone is insufficient to learn LRYGB.

## Introduction

Obesity is increasing rapidly worldwide [1,2]. Bariatric surgery is the most effective method in treating obesity, as it reduces obesity-related comorbidities and improves the quality of life [3-5]. For this reason, laparoscopic bariatric surgery procedures are becoming more common day by day. Laparoscopic Roux-en-Y gastric bypass (LRYGB) and laparoscopic sleeve gastrectomy (LSG) surgeries are the most frequently performed bariatric surgery procedures today. LRYGB has been shown to effectively treat morbid obesity and type 2 diabetes in the short term. Studies show that it causes more weight loss than gastric sleeve surgery in the long run [6]. LRYGB is a more complex procedure than LSG due to a longer learning curve and lack of technical standardization [7].

As in all fields, the popularity of YouTube has increased in the medical world, and with the development of online media streaming, surgeons have also shown interest in the resource [8]. Additionally, some surgeons complete learning curves on electronic media such as YouTube to develop personal skills [9-11]. In the rapidly growing information age, a book published five years ago may be considered old. Still, the most up-to-date information can always be found in the online media stream. Thus, interest in visual and audio media increased instead of reading magazines and books.

The number of medical videos on the internet is increasing day by day. However, learning only from videos without the guidance of a tutorial is worrisome [12,13].

LRYGB is a technically challenging procedure compared to LSG, involving two anastomoses. It is not done as often as LSG [7]. There is consensus in the Netherlands that this metabolic surgery should be performed safely [14]. The key steps for safe LRYGB have been determined in the Delphi consensus.

This study used the key steps of the Delphi consensus to evaluate and determine the value of publicly available LRYGB videos on YouTube for educational surgery.

## Materials and methods

In February 2024, three different searches were done in the search bar of the YouTube platform with the terms "laparoscopic gastric bypass," "laparoscopic Roux-en-Y gastric bypass," and "laparoscopic RYGB." All searches were filtered as videos longer than 20 minutes. The sorting setting remained relevant. The first 50 videos in each search were evaluated. Animations, lectures, advertisements, non-English language videos, and non-surgical videos (preoperative, postoperative vlog, etc.) were excluded from the study.

The videos were evaluated by two surgeons experienced in bariatric surgery. The reliability of surgery in the videos was measured using Delphi Consensus key steps (Table 1). In addition, the videos' upload year, country, resolution, ranking, number of views, comments, and likes and dislikes were evaluated.

**Table 1:** Key steps for laparoscopic Roux-en-Y gastric bypass

Operative setup
Starting of laparoscopy
Creation of the gastric pouch
Creation of biliopancreatic limb
Gastro-jejunal anastomosis
Creation of alimentary limb
Entero-enteral anastomosis
Finishing the bypass
Finishing the operation

The quality of educational content in the videos was measured using the Global Quality Scale (GQS). A modified DISCERN score was also applied to examine the videos' reliability and quality dimensions (Table 2).

**Table 2:** The Global Quality Scale and the modified DISCERN scoring system

The Global Quality Scale (GQS) assigns scores from 1 (indicating poor quality) to 5 (meaning excellent flow and quality).	The modified DISCERN uses a scoring mechanism in which 1 point is awarded for each "Yes" and 0 points for each "No"
The video exhibits poor quality, lacks a coherent structure, lacks essential information, and provides minimal patient benefit. Score: 1	Does the video demonstrate clarity, brevity, and understandability?
The video is generally of below-average quality and lacks proper presentation. While some information is included, many important aspects are missing, resulting in limited patient value. Score: 2	Is the video based on reliable sources of information? (e.g., quotes from broadcasts featuring expert speakers)
Although the quality of the video is average, its flow could be improved. While some critical information is adequately covered, other aspects are inadequately protected, resulting in moderate usefulness for patients. Score: 3	Does the information provided maintain a balanced and unbiased perspective?
The video exhibits commendable quality with a smooth flow. It effectively covers most relevant information, but some topics are left unaddressed. Video is proving valuable to patients. Score: 4	Are additional sources of information provided for patient reference?
The video stands out with exceptional quality and uninterrupted streaming, significantly benefiting patients. Score: 5	Are any areas of uncertainty or debate acknowledged?

Statistical analysis

Analytical assessments were carried out to gain insights into the general features of the study group. The Kolmogorov-Smirnov test was employed to determine if the distributions of numerical variables followed a regular pattern. Subsequently, the independent sample t-test, Mann-Whitney U, and Kruskal-Wallis tests were utilized to contrast the numerical variables across various groups. The Mann-Whitney U test assessed the significance of pairwise differences, incorporating Bonferroni correction to handle multiple comparisons. Numerical variables were displayed as either mean ± standard deviation or median [minimum-maximum]. Categorical variables were compared using the Chi-Square test and were represented as count and percentage. The results were assessed with a 95% confidence interval, where p<0.05 values were deemed statistically significant. The analyses were executed using IBM SPSS Statistics for Windows, Version 23 (Released 2015; IBM Corp., Armonk, New York, United States).

## Results

**Figure 1:**
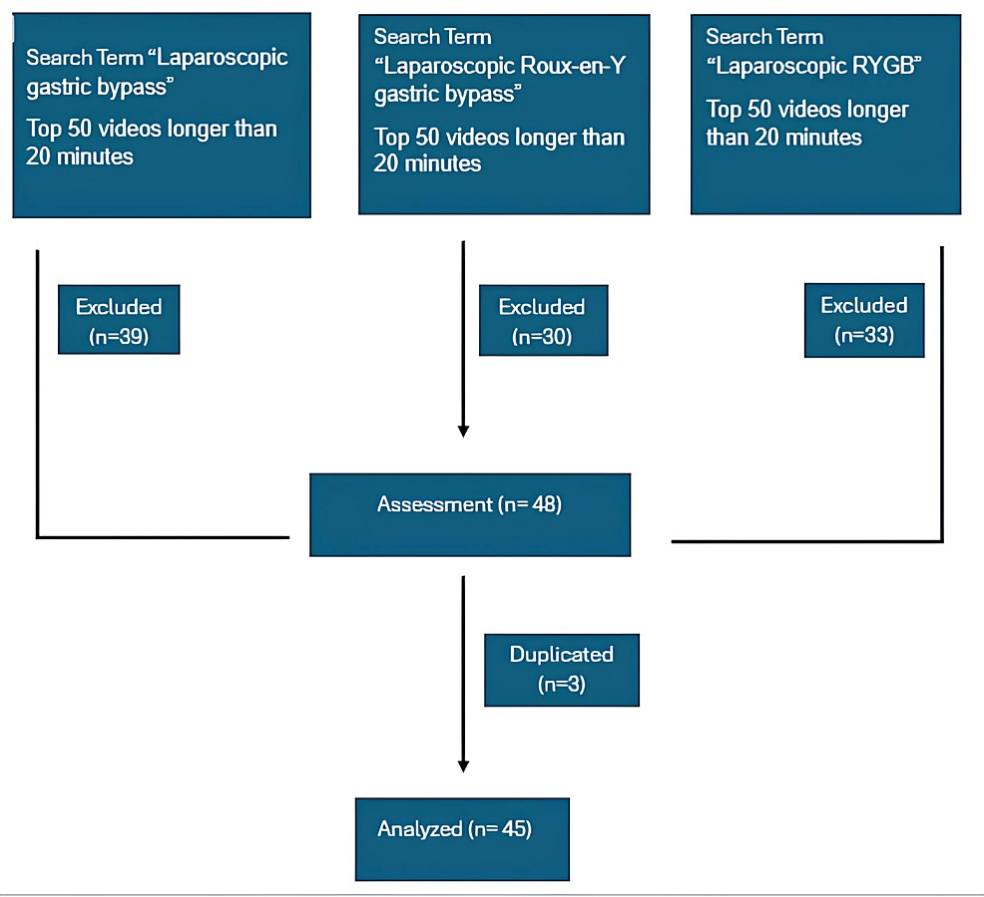
Videos included and excluded from the study

Forty-five videos were included in the evaluation (Figure 1). The country with the highest number of videos was the USA (26 videos), followed by India (10 videos), Brazil (three videos), Turkey (one video), UK (one video), Australia (one video), Luxembourg (one video), Singapore (one video), and Sweden (one video). The average rank of the videos was 25.3±13.1. While 23 (51.2%) of the videos had audio/text narration, 22 (48.8%) did not. The median year of upload of the videos was 2019 (2014-2023). The median duration of the videos, number of views, number of likes, number of dislikes, number of comments, GQS, and modified DISCERN were 40 mins (20-162 mins), 287 (17-106331), 8 (0-1200), 0 (0-9), 0 (0-178), 3 (2-5), and 3 (1-5) respectively. While 14 (31.1%) of these videos were classified as reliable, 31 (68.8%) were not found reliable. Videos found to be reliable are present in [15-28]. Reliable and unreliable videos are compared in Table 3. The proportion of trustworthy videos was not significantly different in all three search categories.

**Table 3:** Comparison of reliable and unreliable videos

		Unreliable (n=31)	Reliable (n=14)	p-value
Rank		24.03±14.6	28.214±8.85	0,329*
Audio/Text Description	Absence	19(%86.4)	3(%13.6)	0,023**
	Present	12 (%52.2)	11(%47.8)	
Upload Year		2018.84±2.79	2018.929±2.52	0,919*
High Definition (HD)	Absence	19 (90.5%)	2 (9.5%)	0.004**
	Present	12 (50%)	12 (50%)	
Duration		43.0 (20-162)	33.5 (20-90)	0,215***
Number of Views		287 (17-5024)	323.5 (110-106331)	0,281***
Like		8 (0-48)	5 (1-1200)	0,853***
Dislike		0 (0-12)	0 (0-178)	0.502***
Number of Comments		0 (0-12)	0 (0-178)	0.787***
Body Mass Index	Absence	26 (66.7%)	13 (33.3%)	0.648**
	Present	5 (83.3%)	1 (16.7%)	
Comorbidity	Absence	26 (70.3%)	11(29.7%)	0.689**
	Present	5 (62.5%)	3 (37.5%)	
Global Quality Scale (GQS)		2 (2-5)	4 (2-5)	0.017***
Modified DISCERN		1 (1-5)	4 (1-5)	0.025***
* Independent Samples T-Test				
** Chi-Square Test				
*** Mann Whitney-U Test				

## Discussion

Training in the digital environment has been increasing rapidly in recent years due to accessibility and cost. Especially during the COVID period, online education became more used due to quarantine and travel restrictions [29,30]. Reasons such as the restriction of elective surgeries and the allocation of a significant part of the budget to the COVID pandemic have increased the tendency towards online platforms due to the deficit in face-to-face applied education in medical education. However, the lack of a tutorial in the online environment and the absence of an identification indicating the videos' accuracy call into question online platforms' reliability. Bariatric surgery videos are also among the frequently encountered videos on online platforms. This can be attributed to the increasing popularity of bariatric surgery. LRYGB is one of the most complex procedures of bariatric surgery. There are many surgery videos about LRYGB on YouTube. To evaluate the reliability of videos, we have covered seven essential steps for LRYGB. LRYGB is technically challenging, and technical variations depend on the surgeon. The Delphi consensus determined nine key steps and 73 sub-steps to standardize this [14]. Since the operation setup and termination steps are not shown in almost any of the videos, we evaluated it over seven steps. Two experienced surgeons evaluated all videos. The most significant factors that made the videos unreliable were that the trocar locations and the jejunum measurement, starting from the traits ligament, were not shown because trocar placement in LRYGB is even more critical than other laparoscopic surgeries. Two anastomoses are performed in different areas. Incorrect placement of ports will negatively affect the surgery. Measurement of the jejunum by advancing clockwise from Treitz is the step that varies the most among surgeons. However, in 22 of the videos (48.2%), they did not show the measurement of its distance from Treitz, but only while performing the anastomosis.

A significant portion of the videos were from the USA. Maybe this is due to the high obesity rate in the USA [31]. The quality and reliability of online medical videos have been addressed recently. YouTube videos, which contain information about health problems for patients, were evaluated for quality. A large amount of misinformation and low-quality videos have been reported [32,33]. 

Our findings showed that most online LRYGB videos are unreliable. We even found that unreliable videos were ranked higher as in had more sort by relevance, although there was no significant difference. Contrary to popular belief, the average duration of reliable videos is shorter than that of unreliable videos, although there is no significant difference. Even with no reliability criteria, the patient did not provide information about their body mass index and comorbidities in most videos. However, these are part of education. Other studies have also revealed that surgical videos on the YouTube platform are unreliable [34,35]. In fact, the study conducted by Ferhatoglu et al. determined that even though healthcare professionals uploaded videos, the videos were below the expected quality even on the WebSurg platform [34]. We believe this is because reviewers or professionals do not evaluate the uploaded videos.

The audio/text comment ratio, high definition (HD) resolution quality, GQS, and modified DISCERN ratio were higher in reliable videos. Bernard et al. used GQS to measure the educational value of videos [36]. A test called DISCERN was developed to evaluate the quality of written information about treatment options for patients and providers. Modified DISCERN is also used in the literature to examine videos' reliability and quality dimensions [37,38]. It is not surprising that these two were detected higher in reliable videos. Many other studies have compared GQS and DISCERN scores between patient-uploaded and healthcare professional-uploaded videos [39-41]. However, in our study, healthcare professionals only uploaded the videos. However, reliable videos had higher GQS and modified DISCERN scores.

Limitations of the study

Our study has some limitations. All searches were made based on relevance for videos longer than 20 minutes on YouTube. Search results may be affected by engagement, quality, and relevance, which may vary between users. Two experienced surgeons performed our evaluations. However, assessments carried out by different experts may yield potentially different results.

## Conclusions

In our study, we examined the reliability of the videos on YouTube of LRYGB, a complicated bariatric surgery, and the quality of the shared information. We found more unreliable videos, and the information shared is insufficient. We concluded that YouTube alone is inadequate for learning LRYGB.
